# Rituximab as the first-line treatment in newly diagnosed systemic lupus erythematosus

**DOI:** 10.3389/fimmu.2025.1599473

**Published:** 2025-06-20

**Authors:** Haiting Wang, Liling Zhao, Shaoying Yang, Huihua Ding, Wanlong Wu, Liqin Yu, Jiajia Jin, Nan Shen, Qiong Fu, Shuang Ye

**Affiliations:** ^1^ Department of Rheumatology, Renji Hospital, Shanghai Jiao Tong University School of Medicine, Shanghai, China; ^2^ Immune Regulation Unit, National Institute of Dental and Craniofacial Research (NIDCR), National Institutes of Health (NIH), Bethesda, MD, United States; ^3^ Suzhou Kowloon Hospital, Shanghai Jiao Tong University School of Medicine, Suzhou, China

**Keywords:** systemic lupus erythematosus, rituximab, newly diagnosed, disease activity, B cell

## Abstract

**Objectives:**

Rituximab (RTX) has been commonly used for the treatment of patients with severe or refractory systemic lupus erythematosus (SLE), yet real-world data concerning RTX as the first-line treatment in newly diagnosed moderate-to-severe SLE patients is lacking.

**Methods:**

We conducted a retrospective cohort study using a newly diagnosed (<3 months) hospitalized Systemic Lupus Inception Cohort (hSLIC) at our center between April 1, 2013 and September 1, 2022. All patients were followed up for at least 12 months or until death. The cohort included patients on RTX (*n* = 104) as the first-line treatment and those on conventional immunosuppressants (IS) (*n* = 154) as comparators. Propensity-score-based inverse probability of treatment weighting (IPTW) was used to minimize possible confounding factors. The primary outcome analyses included attainment of modified lupus low disease activity state (mLLDAS) and remission by 12 months. The secondary outcomes focused on mortality, major flare rates, and the incidence of adverse events of interest, i.e., major infections.

**Results:**

After IPTW, 76.0%/50.5% of RTX-treated patients achieved mLLDAS/remission versus 45.8%/9.7% in the conventional IS group during 12 months of follow-up, respectively (*p* = 0.005 and *p* < 0.001). The sensitivity analyses with renal or neuropsychiatric lupus removal and timeline breakout (pre- versus post-November 2019) confirmed the robustness of RTX’s efficacy in achieving mLLDAS and remission outcomes. Additionally, the incidence of major infections was similar between the two groups (12.5% vs. 8.4%, *p* = 0.288).

**Conclusions:**

In patients with newly diagnosed moderate-to-severe SLE, upfront treatment with RTX was associated with improved clinical outcomes compared to conventional immunosuppressive therapy in terms of achieving low disease activity or remission by 12 months.

## Introduction

Systemic lupus erythematosus (SLE) is a B-lymphocyte-centered autoimmune disorder characterized by significant clinical heterogeneity and the involvement of multiple organ systems (1). Rituximab (RTX), a chimeric monoclonal antibody targeting CD20 on B cells, lies in its mechanism of action to deplete circulating B cells through complement-mediated lysis and antibody-dependent cellular cytotoxicity. RTX has been considered a therapeutic option for treating SLE for over two decades (2, 3). Despite the failure of key randomized controlled trials to meet their primary endpoints (4, 5), clinicians continue to administer RTX based on observational data showing efficacy in both renal and non-renal disease (6, 7). With recommendations in pivotal guidelines (8), RTX remains to be a viable option in relapsing and refractory SLE patients.

With the emerging next-generation anti-CD20 monoclonal antibody (obinutuzumab) and CD19 chimeric antigen receptor T cell (CAR T) therapy, the concept of deeper B cell depletion and “immune reset” has been shaped as the key to better tame or even “cure” SLE. Thus, treating earlier with B cell depletion therapy instead of reserving it as a second- or third-line option to better achieve low disease activity (LDA)/remission before damage accrual ensues is a very appealing strategy in alignment with the key concept (9). However, current evidence supporting this “treat earlier strategy” is short in supply. Only two case series have shown that RTX is effective in newly diagnosed SLE patients, offering a superior steroid-sparing effect compared to standard immunosuppression (10, 11).

Therefore, this study aimed to evaluate the efficacy of RTX as the first-line treatment in a cohort of newly diagnosed moderate-to-severe SLE patients in a real-world setting, focusing on its impact on the attainment of LDA/remission by 12 months.

## Materials and methods

### Patients and study design

This was a retrospective cohort study involving patients newly diagnosed (<3 months) with SLE, who were part of the previously reported hospitalized Systemic Lupus Inception Cohort (hSLIC) (12) from the rheumatology department of Renji Hospital and were enrolled in the study from April 2013 to September 2022. All patients met the 1997 ACR and/or 2019 EULAR/ACR classification criteria (13, 14) for SLE and were followed for at least 12 months or until death. Upon enrollment, baseline data were documented, including demographic details, clinical features, laboratory parameters, treatments, and the Systemic Lupus Erythematosus Disease Activity Index 2000 (SLEDAI-2K) scores (15). Data on subsequent follow-ups and treatment regimens, such as prednisone dosage and immunosuppressive agents, were collected retrospectively.

**Figure 1:**
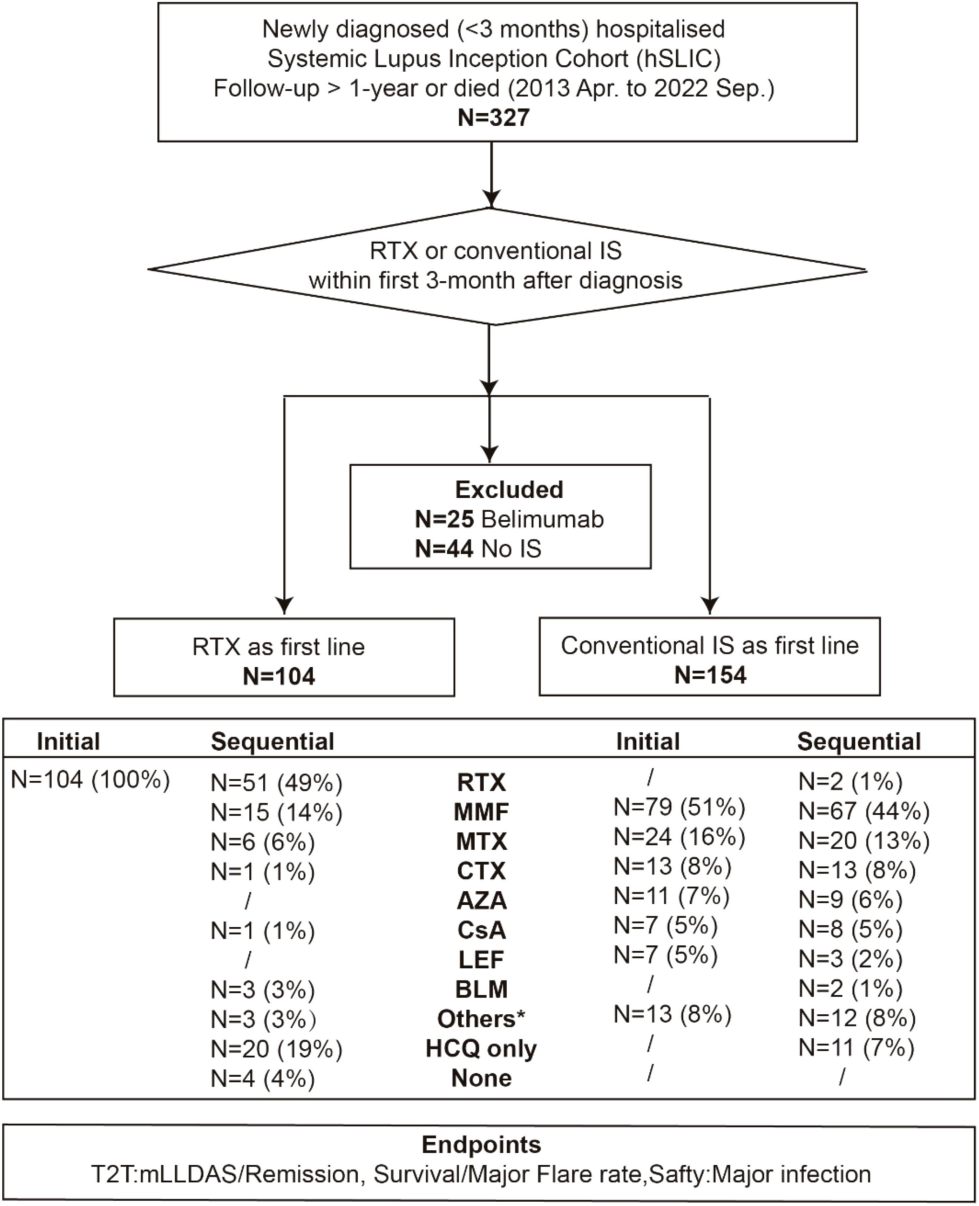
Flow chart of the study. Data are number (%) of patients. RTX, rituximab; IS, immunosuppressants; MMF, mycophenolate mofetil; MTX, methotrexate; CTX, cyclophosphamide; AZA, azathioprine; CsA, cyclosporine A; LEF, leflunomide; BLM, belimumab; Pred, prednisone; HCQ, hydroxychloroquine; T2T, treat-to-target; mLLDAS, modified lupus low disease activity state. The asterisk refers to other IS including tripterygium glycoside, tacrolimus, thalidomide and tofacitinib.

The study cohort was bifurcated based on the use of RTX or conventional immunosuppressants (IS) as the first-line therapy within the first 3 months after diagnosis, resulting in the RTX group and the IS group. The following endpoints were compared between the two groups over a 12-month period: treat-to-target (T2T) outcomes, mortality, major flare rates, and the incidence of major adverse events. Notably, patients who received other B-cell-targeting therapies, such as belimumab, within the first 3-month period were excluded (**Figure 1**). The study protocol was approved by the ethics committee of Renji Hospital (KY2021-059-B).

T2T outcomes: Modified lupus low disease activity status (mLLDAS) and clinical remission on treatment were adapted to evaluate the treatment efficacy (16–18). Briefly, mLLDAS encompassed a SLEDAI-2K score of ≤4, without any activity in major organ systems or new features of disease activity compared with the previous assessment, and on a stable regimen of prednisone of ≤7.5 mg/day and maintenance doses of immunosuppressive medications at the visit date. Clinical remission on treatment was defined as clinical SLEDAI-2K = 0 and prednisone of ≤5 mg/day and immunosuppressive drugs at maintenance dose at the visit date. Physician global assessment (PGA) was not included in either definition (19).

Major flare: A major flare was defined (20–22) by the occurrence of at least one of the following criteria: (1) an increase in SLEDAI-2K score by more than 12 points, (2) a doubling of the prednisone dosage or an increase to over 0.5 mg/kg/day, (3) hospitalization due to a SLE flare, or (4) upgrading IS regimen because of uncontrolled disease.

Major infections: Common Terminology Criteria for Adverse Events (CTCAE) was used to grade infections (https://ctep.cancer.gov/protocoldevelopment/electronic_applications/docs/CTCAE_v5_ Quick_ Reference_5x7.pdf). Major infection (CTCAE grade 3 or higher) was characterized by a diagnosis that was either microbiologically or clinically established and was treated with intravenous antimicrobials (12, 23).

### Statistical analysis

Patient characteristics were presented as mean (standard deviation) for continuous variables or as numbers and percentages for categorical variables. Between-group comparisons were conducted using Student’s *t*-test for normally distributed variables and Mann–Whitney *U*-test for non-normally distributed variables, while categorical variables were analyzed using Fisher’s exact test. Survival rates were evaluated using the Kaplan–Meier method. To account for potential baseline differences between the groups, propensity-score-based inverse probability of treatment weighting (IPTW) with stabilized weights was applied. We calculated the standardized mean differences (SMDs) for each covariate and when the SMD was 0.2 or less after IPTW, the confounder was considered to have no between-group difference. Outcomes were comparable where there were no between-group differences in all covariates. Additionally, the last observation carries forward (LOCF) method was employed to address the missing data in the endpoint assessment by 12 months (24, 25). All *P*-values reported were two-sided, with statistical significance defined as *P* < 0.05. Statistical analyses were conducted using R (version 4.4.1) and GraphPad Prism (version 9.0).

## Results

### Baseline characteristics of the whole cohort

**Figure 2:**
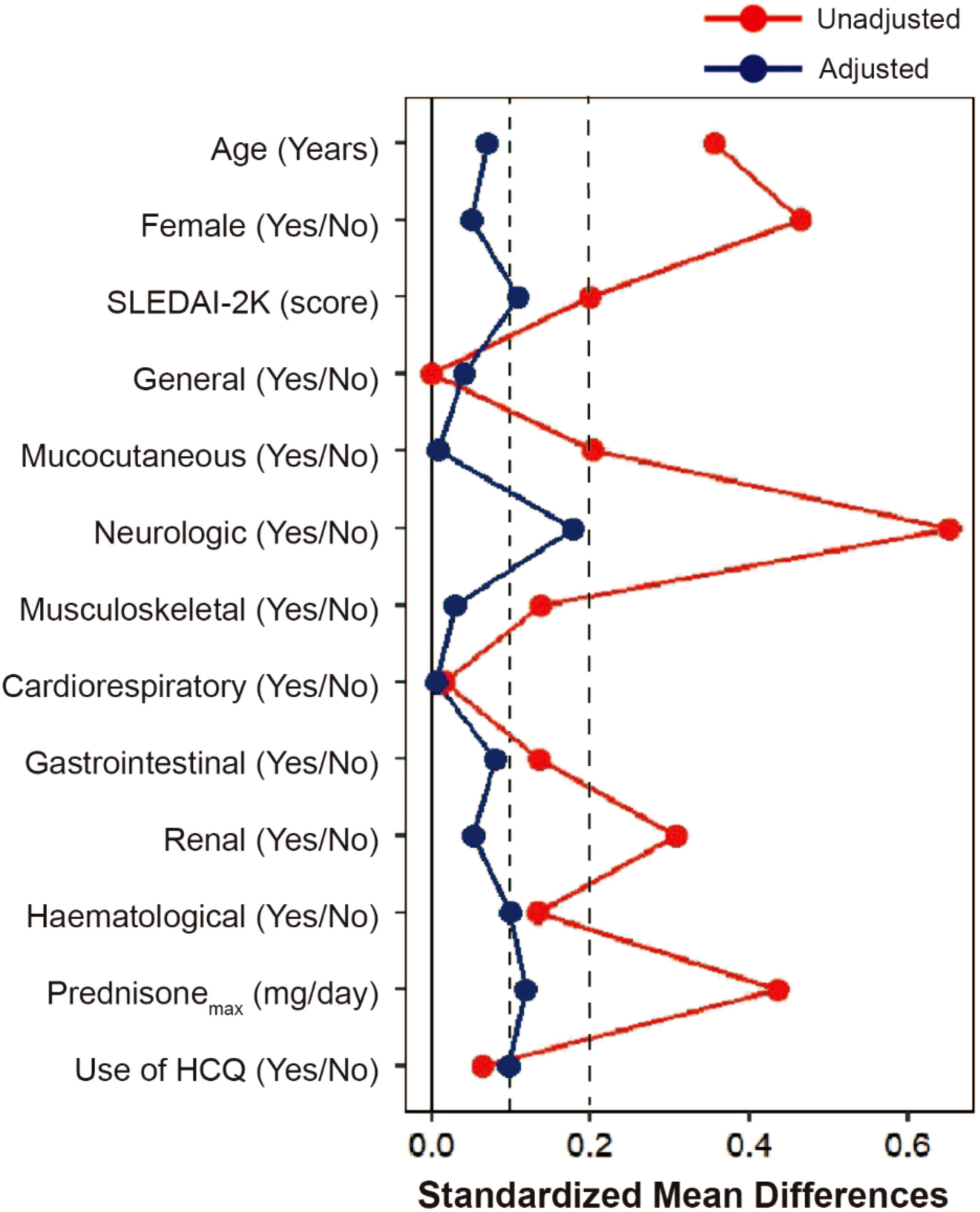
Covariate balance before and after adjusting. Love plot with absolute standardized mean differences (SMD) between rituximab and conventional immunosuppressant groups for a subset of covariates before IPTW (Unadjusted) and after IPTW (Adjusted). When the SMD was 0.2 or less after IPTW, the confounder was considered to have no between-group difference. SLEDAI-2K, Systemic Lupus Erythematosus Disease Activity Index 2000; IPTW, inverse probability of treatment weighting.

From April 2013 to September 2022, 258 newly diagnosed SLE patients were included in the study, with a median time from diagnosis to enrollment of 7 days (range, 0–92 days). Among these, 104 patients received RTX as a first-line treatment, while 154 were treated with conventional IS (**Figure 1**). The baseline characteristics of each group are summarized in **Table 1**; **Supplementary Tables S1**, **S2**. The mean (SD) SLEDAI-2K score was 13.79 (7.72) for the RTX group, compared to 12.44 (5.68) in the conventional IS group. In the RTX group, the average time from hospital admission to receiving the RTX infusion was 8.5 days, with four patients having received the infusion prior to admission. Among the cohort, 49.0% (51/104) received three infusions (with the third infusion as a “booster” at 6 months apart), 44.2% (46/104) received two infusions, and 6.7% (7/104) received only one infusion, with the average cumulative dosages of 900 mg (200–1,500 mg) during 12 months. Patients receiving RTX were younger at SLE onset and had a lower percentage of male patients than those in the conventional IS group. Neuropsychiatric involvement was more common in the RTX group, whereas lupus nephritis was more frequent among those receiving conventional IS. Moreover, patients in the RTX group received a higher maximum prednisone dosage after diagnosis. After adjusting for confounding factors including age, sex, SLEDAI-2K score, British Isles Lupus Assessment Group (BILAG) domain involvement (26), low complement 3 levels, presence of anti-dsDNA antibodies, antiphospholipid antibodies (APL), maximum prednisone dosage, and exposure of hydroxychloroquine using IPTW (**Figure 2**), the baseline discrepancies were evened out (**Table 1**).

**Table 1:** Patient characteristics in the rituximab and conventional immunosuppressant groups before and after IPTW.

Variables	Before IPTW		*P*-value	After IPTW		*P*-value
	Rituximab	Conventional IS		Rituximab	Conventional IS	
	*n* = 104	*n* = 154		*n* = 104	*n* = 192	
Demographic characteristics						
Age (years)	32.4 (13.2)	37.25 (13.8)	**0.005**	30.21 (14.12)	33.13 (13.19)	0.424
Gender, *n* (%female)	102 (98.1)	132 (85.7)	**0.002**	80.9 (77.9)	176.8 (92.0)	0.219
Clinical manifestations						
SLEDAI-2K score	13.79 (7.72)	12.44 (5.68)	0.106	12.35 (6.31)	13.52 (6.31)	0.363
BILAG category						
General (%)	48 (46.2)	71 (46.1)	1	35.5 (34.2)	68.0 (35.4)	0.909
Mucocutaneous (%)	53 (51.0)	94 (61.0)	0.14	44.7 (43.1)	85.2 (44.4)	0.917
Neuropsychiatric (%)	27 (26.0)	6 (3.9)	**<0.001**	13.2 (12.7)	54.4 (28.3)	0.102
Musculoskeletal (%)	26 (25.0)	48 (31.2)	0.35	22.1 (21.3)	42.0 (21.9)	0.938
Cardiorespiratory (%)	64 (61.5)	96 (62.3)	1	71.0 (68.3)	117.9 (61.3)	0.537
Gastrointestinal (%)	9 (8.7)	8 (5.2)	0.399	7.0 (6.8)	18.6 (9.7)	0.608
Renal (%)	27 (26.0)	62 (40.3)	**0.025**	45.2 (43.5)	60.4 (31.5)	0.383
Hematological (%)	29 (27.9)	34 (22.1)	0.359	21.0 (20.2)	50.7 (26.4)	0.477
Serological markers						
Low complement 3 (g/L)	88 (84.6)	130 (84.4)	1	91.6 (88.2)	170.0 (88.5)	0.943
Anti-dsDNA (%)	73 (70.2)	108 (70.1)	1	75.9 (73.0)	108.0 (56.2)	0.144
APL (%)	19 (18.3)	23 (14.9)	0.589	16.9 (16.3)	36.6 (19.1)	0.759
Treatment						
Prednisone_max_ (mg/day)	189 (188)	120 (123)	**<0.001**	140 (149)	173 (170)	0.443
Methylprednisolone pulse (%)	17 (16.3)	11 (7.1)	0.033	10.9 (10.8)	36.6 (20.4)	0.284
Use of hydroxychloroquine (%)	94 (90.4)	142 (92.2)	0.774	97.6 (93.9)	172.1 (89.6)	0.41

Data are mean (SD) or number (%) of patients.

IPTW, inverse probability of treatment weighting; SLEDAI-2K, Systemic Lupus Erythematosus Disease Activity Index 2000; BILAG, British Isles Lupus Assessment Group; IS, immunosuppressants; APL, antiphospholipid antibody; Prednisone_max_ (mg/day), the highest daily dose of intravenous methylprednisolone administered during the initial hospitalization period; Methylprednisolone pulse (%), proportion of patients who received intravenous methylprednisolone pulse therapy, defined as ≥500 mg/day for 3 to 5 consecutive days, during the initial hospitalization period. Bold values indicate statistical significance (p < 0.05).

### Treatment efficacy outcomes

**Figure 3:**
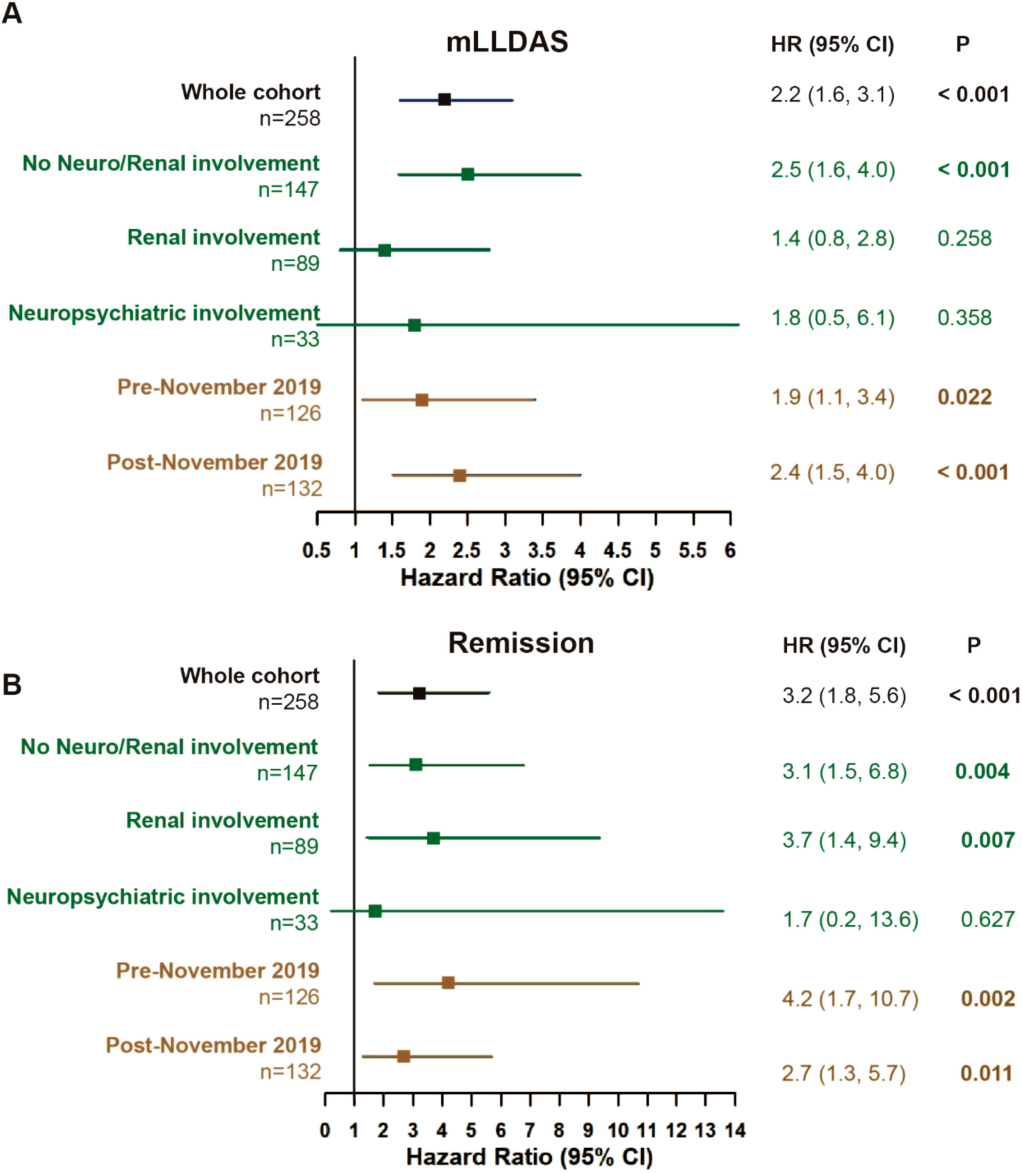
Treat-to-target outcomes. Attainment of mLLDAS **(A)** and remission **(B)** within 12 months in the whole cohort and sensitivity analyses (rituximab versus conventional immunosuppressants group). The model was adjusted for age, gender, and maximum prednisone dosage. Pre-November 2019 (no COVID-19 exposure), patients who completed their 12-month follow-up before November 2019. Post-November 2019 (COVID-19 exposure), patients who completed their 12-month follow-up after November 2019; CI, confidence interval; mLLDAS, modified lupus low disease activity state; SLEDAI-2K, Systemic Lupus Erythematosus Disease Activity Index 2000; Neuro, neuropsychiatric; HR, hazard ratio. At 12 months, follow-up data were missing for 12 out of 258 patients (4.6%) (three in the RTX group and nine in the conventional IS group). The last observation carries forward (LOCF) method was employed to address the missing data in the endpoint assessment by 12 months.

By 12 months, 72 (69.2%) patients in the RTX group and 72 (46.8%) in the conventional IS group achieved mLLDAS, while remission was attained by 35 patients (33.7%) in the RTX group compared to 23 patients (14.9%) in the conventional IS group (*p* < 0.001 and *p* = 0.001). After applying IPTW, the proportions of patients achieving mLLDAS and remission increased to 76.0% and 50.5% in the RTX group, respectively, compared to 45.8% and 9.7% in the conventional IS group (*p* = 0.005 and *p* < 0.001) (**Table 2**). Time-to-event analysis further demonstrated that the patients in the RTX group achieved both mLLDAS and remission significantly earlier than those in the conventional IS group (**Supplementary Figure S1**). RTX induced a substantial decline in peripheral CD19^+^ B cell counts by 6 months, though the residual B cells remained detectable (median 30/μL). Partial reconstitution was observed by 12 months (**Supplementary Figure S2**). Sensitivity analyses with neuropsychiatric or renal-involved patients removed or timeline breakout (pre-November 2019 versus post-November 2019, taking the COVID-19 pandemic into consideration) all yielded the robustness of RTX superiority in terms of mLLDAS/remission attainment (**Figures 3A, B**). However, probably due to the underpowered small number of patients, the effects of RTX on neuropsychiatric SLE (NPSLE) was non-significant. Additionally, by using renal partial/complete remission (PR/CR) as endpoints, no difference in PR/CR between RTX and conventional IS can be appreciated in the lupus nephritis subgroup (**Supplementary Table S3**).

**Table 2:** Treatment efficacy outcomes at 12 months.

Outcomes	Before IPTW		*P*-value	After IPTW		*P*-value
	Rituximab	Conventional IS		Rituximab	Conventional IS	
	*n* = 104	*n* = 154		*n* = 104	*n* = 192	
mLLDAS*	72 (69.2)	72 (46.8)	<0.001	79.0 (76.0)	88.1 (45.8)	0.005
SLEDAI-2k ≤ 4	97 (93.3)	120 (77.9)	0.002	99.7 (95.9)	133.1 (69.3)	<0.001
Pred ≤7.5 mg/day	75 (72.1)	75 (48.7)	<0.001	81.5 (78.4)	90.4 (47.1)	0.003
Remission*	35 (33.7)	23 (14.9)	0.001	52.5 (50.5)	18.6 (9.7)	<0.001
cSLEDAI-2k=0	68 (65.4)	73 (47.4)	0.007	77.7 (74.8)	74.7 (38.9)	0.001
Pred ≤ 5mg/day	51 (49.0)	36 (23.4)	<0.001	64.1 (61.7)	45.7 (23.8)	0.004
**All-cause deaths**	1 (1.0)	7 (4.5)	0.100	1 (1.0)	15 (7.8)	0.120
**Major flares**	10 (9.6)	30 (19.5)	0.027	7 (6.7)	36 (18.8)	0.221

Data are number (%) of patients.

IPTW, inverse probability of treatment weighting; cSLEDAI-2K, clinical Systemic Lupus Erythematosus Disease Activity Index 2000; mLLDAS, modified lupus low disease activity state; IS, immunosuppressants; Pred, prednisone.

*At 12-month, follow-up data were missing for 12 out of 258 patients (4.6%) (3 in the RTX group and 9 in the conventional IS group). The last observation carries forward (LOCF) method was employed to address the missing data in the endpoint assessment by 12-month.

The 1-year overall survival rates were comparable between the RTX and conventional IS groups (99.0% vs. 92.2%, *p* = 0.120 after IPTW) (**Table 2**). Infection was identified as the leading cause of mortality (**Supplementary Table S4**). Notably, the RTX group exhibited a significantly higher major flare-free survival rate at 12 months compared to the IS group (before IPTW adjustment: 90.4% vs. 80.5%, *p* = 0.027); however, this difference was no longer significant after IPTW adjustment (**Table 2**). The most frequently observed major flare was the onset or exacerbation of lupus nephritis (**Supplementary Table S4**).

### Adverse events

In the RTX group, a total of seven patients (6.7%) experienced infusion-related adverse reactions, including skin rash and fever (**Table 3**). Additionally, 13 patients (12.5%) had one or more major infectious complications during follow-up, predominantly respiratory and skin/soft tissue infections (**Table 3**). A comparable incidence of major infections was documented among conventional IS group (13/154, 8.4%, *p* = 0.288).

**Table 3:** Summary of adverse events.

Events	Rituximab *n* = 104	Conventional IS *n* = 154	*P*-value
Infusion-related reaction	7 (6.7)	/	
Major Infection	13 (12.5)	13 (8.4)	0.288
Pneumonia	6 (5.8)	7 (4.5)	0.659
Skin and soft-tissue infections	4 (3.8)	4 (2.6)	0.570
Bloodstream infections	2 (1.9)	1 (0.6)	0.349
Urinary tract infections	1 (1.0)	0	0.223
Central nervous system infections	0	1 (0.6)	0.410

Data are number (%) of patients.

## Discussion

This study is intended to evaluate RTX as a first-line treatment for newly diagnosed moderate-to-severe SLE in a real-world setting. By utilizing propensity score-based IPTW, we have demonstrated that RTX showed superior efficacy in achieving LDA and remission. These findings highlight the value of RTX as an upfront therapeutic option for newly onset active SLE.

The LUNAR and EXPLORER trials, which focused on lupus nephritis and extrarenal lupus, respectively, failed to achieve their primary endpoints (4, 5). Notably, these studies enrolled patients with long-standing disease durations (i.e., approximately 2-year-old lupus nephritis in the LUNAR trial and 8-year-old SLE in the EXPLORER trial). In contrast, our study included newly diagnosed patients with moderate-to-severe active SLE and major organ involvement, with a median diagnosis-to-enrollment time interval of 7 days; thus, the patients in our cohort were basically treatment-naïve. This, to some extent, explains the high RTX responsiveness in terms of attainment of LDA/remission, which is in line with the notion that the more previous immunosuppressive agent exposures (hence “refractory”), the higher the likelihood of inadequate response to RTX (27, 28). Furthermore, the concept of using biologics early in the disease course has been further supported by recent evidence of the efficacy of initial combination therapy with belimumab, another B-cell-targeted biologics, in newly diagnosed adult or childhood-onset lupus nephritis (29). In this context, our findings, with a favorable safety profile, help to pave the way for a paradigm shift in SLE management, i.e., prioritizing earlier and more targeted interventions to achieve disease modification, which may result in not only short-term LDA/remission attainment but probably also less organ damage in the long term (9).

This study is subjected to several limitations. First and foremost, attributing to the nature of this real-world single-center cohort study design, confounding issues such as referral and selection bias were inevitable. Although with propensity score matching and IPTW adjustments, our study cannot replace a more stringent randomized controlled trial. Second, the study was underpowered to conduct in-depth subgroup analyses such as neuropsychiatric or renal-involved patients, leaving these important SLE domains largely untouched, albeit the signal of RTX as a first-line treatment on newly diagnosed SLE as a whole is significant. Third, the dosing of RTX was determined at the discretion of treating physicians rather than being standardized, along with lack of scheduled B-cell checkup, which limited the ability to evaluate the B-cell dynamics and its relationship with clinical outcomes. Finally, this study did not include an assessment of cumulative organ damage using the SLICC/ACR Damage Index (SDI) (30) due to the inconsistent availability of complete and standardized data across the cohort. We acknowledge this as a limitation and emphasize the importance of incorporating systematic, longitudinal damage evaluations in future prospective studies to more accurately assess the long-term impact of RTX.

In summary, this study provides real-world evidence supporting RTX as a first-line treatment for newly diagnosed moderate-to-severe SLE by demonstrating significant improvements in the attainment of low disease activity or remission in a time frame of 12 months.

## Data Availability

The datasets presented in this study can be found in online repositories. The names of the repository/repositories and accession number(s) can be found in the article/**Supplementary Material**.
