# Supplementary material for: Rituximab as the first-line treatment in newly diagnosed systemic lupus erythematosus
Source: Front Immunol. 2025 Jun 20;16:1599473. doi: 10.3389/fimmu.2025.1599473 (PMC12226576; doi:10.3389/fimmu.2025.1599473)
Supplement: Supplementary file 1 [file Table1.docx]

**Supplementary Table 1. Autoantibody Profiles**

| **Variables** | **Rituximab** | **Conventional IS** | **P value** |
| --- | --- | --- | --- |
|  | **n=104** | **n=154** |  |
| Anti-SSA (%) | 69 (66.3) | 81 (52.6) | 0.028 |
| Anti-SSB (%) | 15 (14.4) | 27 (17.5) | ns |
| Anti-Sm(%) | 37 (35.6) | 46 (29.9) | ns |
| Anti-RNP (%) | 38 (36.5) | 60 (39.0) | ns |
| Anti-CENP-B | 1 (1.0) | 6 (3.9) | ns |
| Anti-Scl70 (%) | 2 (1.9) | 2 (1.3) | ns |
| Anti-Rib-P (%) | 27 (26.0) | 30 (19.5) | ns |
| Anti-Histone (%) | 9 (8.7) | 15 (9.7) | ns |
| Anti-Nucleosome (%) | 25 (24.0) | 47 (30.5) | ns |
| Anti-MPO (%) | 1 (1.0) | 2 (1.3) | ns |
| Anti-PR3 (%) | 3 (2.9) | 1 (0.6) | ns |
| Anti-CCP (%) | 5 (4.8) | 5 (3.2) | ns |
| Anti-G6P (%) | 1 (1.0) | 2 (1.3) | ns |

Data are number (%) of patients.

**Supplementary Table 2. Neuropsychiatric Involvement details**

|  | **Rituximab N=27** | **Conventional IS**  **N=6** |
| --- | --- | --- |
| Lupus headache | 6 (22.2) | 2 (33.3) |
| Recent onset seizure | 6 (22.2) | 1 (16.7) |
| New onset stroke | 8 (29.6) | 0 |
| New onset sensory or motor neuropathy | 5 (18.5) | 1 (16.7) |
| Psychosis | 1 (3.7) | 1 (16.7) |
| Organic brain syndrome | 3 (11.1) | 1 (16.7) |
| Visual disturbance | 1 (3.7) | 0 |
| Acute confusional state | 2 (7.4) | 0 |

Data are number (%) of patients.

**Supplementary Table 3. Baseline characters and renal response in the LN subgroup(n=87) with renal outcomes available at 1 year (n=66)**

|  | **Rituximab**  **n=20** | **Conventional IS**  **n=46** | **P value** |
| --- | --- | --- | --- |
| Female n (%)  Age mean±SD | 20 (100)  31.4±9.8 | 34 (73.9)  36.8±13.6 | **0.012**  0.074 |
| SLEDAI-2K mean±SD | 21.1±7.6 | 15.7±4.8 | **0.007** |
| Proteinuria (g) mean±SD |  |  |  |
| Baseline  1 year | 4.16±6.43  0.84±1.29 | 3.10±3.87  0.79±3.21 | 0.500  0.935 |
| eGFR(ml/min/1.73m^2^) mean±SD |  |  |  |
| Baseline | 66.9±43.5 | 96.7±35.4 | **0.012** |
| 1 year | 86.2±42.7 | 102.6±24.5 | 0.132 |
| Biopsy performed n (%) | 5(25) | 31(67.4) | **0.001** |
| II | 0/5(0) | 3/31(9.6) | 0.468 |
| III | 1/5(20) | 4/31(12.9) | 0.670 |
| IV | 3/5(60) | 9/31(29.0) | 0.173 |
| V | 0/5(0) | 5/31(16.1) | 0.333 |
| III/IV+V | 0/5(0) | 4/31(12.9) | 0.394 |
| VI | 1/5(20) | 0/31(0) | **0.012** |
| TMA reported | 1/5(20) | 1/31(3.2) | 0.129 |
| NA | 0/5(0) | 5/31(16.1) | 0.333 |
| **Renal outcomes n (%)** |  |  |  |
| CR | 10(50.0) | 27(58.7) | 0.513 |
| PR | 1(5.0) | 7(15.2) | 0.242 |
| NR | 9(45.0) | 12(26.1) | 0.130 |
| PERR | 11(55.0) | 33(71.7) | 0.185 |
| ESRD | 3(15.0) | 0(0) | **0.007** |
| Death | 1(5.0) | 3(6.5) | 0.812 |

Data are mean ± SD or number (%) of patients. SLEDAI-2K: Systemic Lupus Erythematosus Disease Activity Index 2000; TMA: thrombotic microangiopathy; NA: not available (renal biopsy performed outside Renji hospital, but results were unavailable from medical records); CR: complete response; PR: partial response; NR: non-response; PERR: primary efficacy renal response rate; ESRD: End-stage renal disease.

Renal pathologies were classified based on the International Society of Nephrology and Renal Pathology Society criteria. CR was defined as 24- hour urine protein<0.5 g and eGFR≥90 mL/min/1.73 m^2^ or stable renal function (no decline greater than 10% from the preflare value) without rescue therapy. PR was defined as a reduction of at least 50% in proteinuria with a maximum 24- hour urine protein≤3 g and eGFR≥90 mL/min/1.73 m^2^ or stable renal function (no decline greater than 10% from the preflare value) without rescue therapy. NR was defined as patients with LN who did not fulfil either CR or PR. PERR was defined as 24- hour urine protein<0.7 g and eGFR≥60 mL/min/1.73 m^2^ or stable renal function (no decline greater than 20% from the preflare value) without rescue therapy. ESRD is a diagnosis determined by the clinician initiating long-term renal replacement therapy.

**Supplementary Table 4. Causes of death and types of major flare during 12 months follow-up**

|  | **Rituximab** | **Conventional IS** |
| --- | --- | --- |
| **Causes of death** | **n=1** | **n=7** |
| Infection | 1 (100) | 6 (85.7) |
| Active disease | 0 | 1 (14.2) |
|  |  |  |
| **Types of major flare** | **n=9** | **n=23** |
| Lupus nephritis | 4 (44.4) | 11 (48.0) |
| Neuropsychiatric SLE | 1 (11.1) | 1 (4.0) |
| Gastrointestinal vasculitis | 0 | 2 (9.0) |
| Systemic disease flare | 4 (44.4) | 9 (39.0) |

Data are number (%) of patients. SLE: Systemic Lupus Erythematosus.

**Supplementary Figure 1. Time to mLLDAS and Remission in RTX versus Conventional IS Groups**


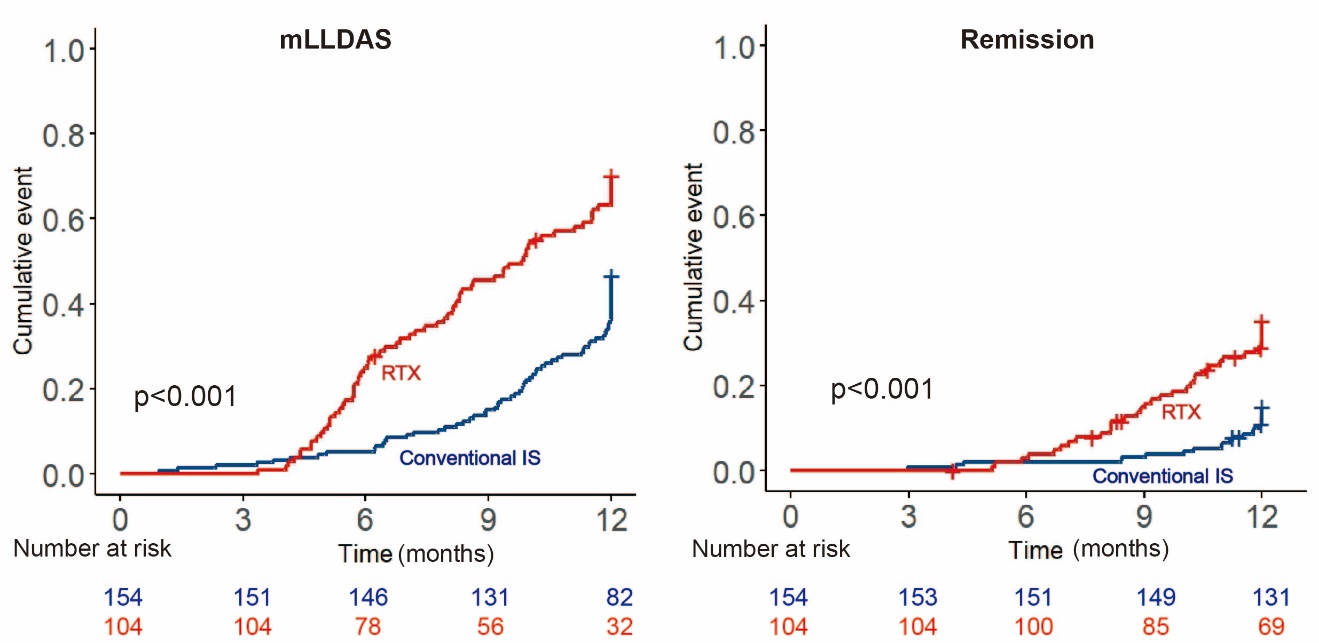


Kaplan-Meier curves showing time to achievement of modified Lupus Low Disease Activity State (mLLDAS, left) and remission (right) over a 12-month period in patients treated with rituximab (RTX) or conventional immunosuppressants (IS). Time is shown in months on the x-axis, and the cumulative proportion of patients achieving each outcome is shown on the y-axis. Log-rank test p-values indicate the statistical difference between the two groups. The number of patients at risk at each time point is listed below the curves.

**Supplementary Figure 2. Longitudinal CD19⁺ B Cell counts following initial Rituximab Treatment**


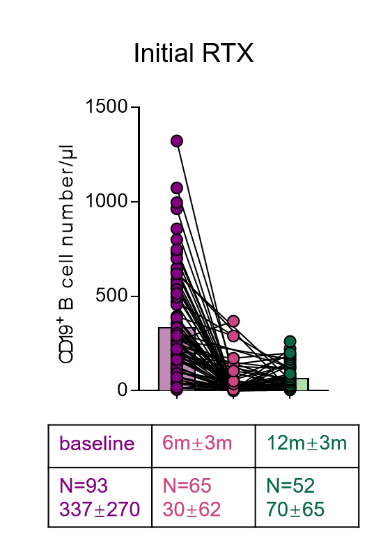


CD19⁺ B cell numbers (cells/μL peripheral blood) were measured at baseline, 6 ± 3 months, and 12 ± 3 months following initial rituximab (RTX) administration. Each dot represents an individual patient. Summary statistics (mean ± SD) and sample sizes (N) at each time point are shown in the table below the graph.
